# Evaluation of the Reactivity and Receptor Competition of HLA-G Isoforms toward Available Antibodies: Implications of Structural Characteristics of HLA-G Isoforms

**DOI:** 10.3390/ijms20235947

**Published:** 2019-11-26

**Authors:** Atsushi Furukawa, Manami Meguro, Rika Yamazaki, Hiroshi Watanabe, Ami Takahashi, Kimiko Kuroki, Katsumi Maenaka

**Affiliations:** 1 Laboratory of Biomolecular Science, Faculty of Pharmaceutical Sciences, Hokkaido University, Sapporo 060-0812, Japan; afuru@pharm.hokudai.ac.jp (A.F.); manami.meg@frontier.hokudai.ac.jp (M.M.); rika@pharm.hokudai.ac.jp (R.Y.); watanabe_2896@eis.hokudai.ac.jp (H.W.); ami.takahashi@frontier.hokudai.ac.jp (A.T.); k-kimiko@pharm.hokudai.ac.jp (K.K.); 2 Center for Research and Education on Drug Discovery, Faculty of Pharmaceutical Sciences, Hokkaido University, Sapporo 060-0812, Japan

**Keywords:** immune checkpoint, HLA-G, antibody, ELISA

## Abstract

The human leucocyte antigen (HLA)-G, which consists of seven splice variants, is a tolerogenic immune checkpoint molecule. It plays an important role in the protection of the fetus from the maternal immune response by binding to inhibitory receptors, including leukocyte Ig-like receptors (LILRs). Recent studies have also revealed that HLA-G is involved in the progression of cancer cells and the protection from autoimmune diseases. In contrast to its well characterized isoform, HLA-G1, the binding activities of other major HLA-G isoforms, such as HLA-G2, toward available anti-HLA-G antibodies are only partially understood. Here, we investigate the binding specificities of anti-HLA-G antibodies by using surface plasmon resonance. MEM-G9 and G233 showed strong affinities to HLA-G1, with a nM range for their dissociation constants, but did not show affinities to HLA-G2. The disulfide-linker HLA-G1 dimer further exhibited significant avidity effects. On the other hand, 4H84 and MEM-G1, which can be used for the Western blotting of HLA-G isoforms, can bind to native HLA-G2, while MEM-G9 and G233 cannot. These results reveal that HLA-G2 has a partially intrinsically disordered structure. Furthermore, MEM-G1, but not 4H84, competes with the LILRB2 binding of HLA-G2. These results provide novel insight into the functional characterization of HLA-G isoforms and their detection systems.

## 1. Introduction

Human leucocyte antigen (HLA)-G is one of the non-classical major histocompatibility complex-I (MHC-I) molecules, a group that includes HLA-E and HLA-F [1]. Unlike classical MHC-I molecules, HLA-G shows restricted tissues expression, such as in the placenta, and some regulatory T cells [2,3]. HLA-G is reportedly not involved in the antigen presentation for stimulating the immune system but binds inhibitory leukocyte immunoglobulin-like receptors (LILR) molecules to suppress immune activation [4]. In the placenta, HLA-G plays a redundant role in the protection of the fetus from maternal immune responses. Notably, the amounts of HLA-G inversely correlate with the severity of autoimmune diseases [5,6,7]. We also demonstrated that the administration of the HLA-G molecule improved the symptoms of the collagen induced arthritis model mice and the atopic dermatitis model mice [8,9,10]. Furthermore, certain tumor cells also express HLA-G on the surface to suppress immune activation and escape immune surveillance [11,12]. Recent studies have exhibited the importance of the interactions of immune checkpoint inhibitory receptors with cognate ligands, such as program cell death protein 1 (PD-1) and PD-1Ls, and cytotoxic cell lymphocyte antigen-4 (CTLA-4) and CD80/CD86 interactions for cancer therapy [13,14,15]. Therefore, HLA-G is a prominent drug candidate for autoimmune diseases, and HLA-Gs-LILR interactions are also one of the targets for cancer immune therapy.

HLA-G comprises seven isoforms (HLA-G1 to G7), which harbor different combinations of α1, α2, and α3 domains of heavy chain and β2-microglobulin (β2m) in extracellular regions, together with the transmembrane and cytoplasmic domain [16]. HLA-G1 is a well characterized molecule among HLA-Gs, and its ectodomain consists of α1-α2-α3 domains, β2m, and a peptide. HLA-G2 also has a membrane-anchoring form and contains only two domains, α1 and α3, with a heavy chain in the extracellular region. Our recent biochemical and electron microscopic studies revealed that the ectodomain of HLA-G2 forms a homodimer via non-covalent interactions and strongly binds to LILRB2 [9,17]. Furthermore, HLA-G2 is considered to play an important role in humans who cannot produce functional HLA-G1 due to an HLA-G–null allele [18,19]. HoWangYin et al. demonstrated that the Fc-fusion version of HLA-G2 can prolong the survival of a mouse with skin allografts [20]. We also showed that HLA-G2 has a significant therapeutic effect against arthritis model mice [9]. HLA-G3 and HLA-G4 are predicted to be expressed in membrane-anchoring forms, and contain only single α1 domain and α1–α2 domains, respectively. HLA-G5, G6, and G7 are soluble forms (non-membrane-anchoring forms) of HLA-G1, G2, and G4, respectively [21]. In addition, the HLA-G1 molecule forms the disulfide-linked HLA-G1 homodimer, which reportedly plays an important role in immune suppression [22,23]. The structural complexity of HLA-G still makes it difficult to precisely evaluate the amount and function of each isoform in vivo. In order to measure HLA-G molecules in the serum of autoimmune and cancer patients, enzyme linked immunosolvent assays (ELISAs) were developed using monoclonal antibodies against HLA-G [24]. MEM-G9 and G233 are widely used in HLA-G ELISA as a capture antibody [25]. MEM-G9 reportedly binds surface expressed HLA-G1 but not HLA-G2, G3, or G4 [26]. Furthermore, the other HLA-G antibody, 4H84, binds denatured or β2m-free HLA-G proteins and is considered to be a useful antibody due to its reactivity in Western blotting against all HLA-G isoforms containing the α1 domain [27,28,29,30,31]. MEM-G1 is also a well-known HLA-G antibody and is reported to recognize the denatured form of the HLA-G protein [32]. However, the molecular specificities of the antibodies against each splice variant and the dimer/monomers of HLA-G remain unclear.

Here, we investigate the reactivities of HLA-G antibodies to HLA-G1 and HLA-G5 (hereafter referred to as HLA- G1 because they have essentially identical ectodomains), and HLA-G2 and HLA-G6 (hereafter referred to as HLA- G2 because they have essentially identical ectodomains). The 4H84 and MEM-G1 are bound to native HLA-G2, not to HLA-G1. Since 4H84 and MEM-G1 can be used for Western blotting, thus indicating that they can recognize denatured HLA-G proteins, HLA-G2 presumably has an unstructured part that is reactive to antibodies. Interestingly, 4H84 can bind to LILRB2-captured HLA-G2, suggesting that the binding sites for 4H84 and LILRB2 are located at sufficiently far distances. This result also suggests the possibility that the combination of 4H84 and LILRB2 enable the development of a sandwich ELISA useful for detection of HLA-G2 and related isoforms. Our biophysical analyses using antibodies revealed the structural features and predicted the ligand binding site of the HLA-G isoforms.

## 2. Results

### 2.1. A Surface Plasmon Resonance (SPR) Analysis of Antibody Binding Towards HLA-G1 and Its Disulfide-Linked Dimer

In order to evaluate the binding ability of the antibodies against native HLA-G1 and its homodimer, we performed an SPR analysis. 3.1–50 nM of HLA-G proteins were injected over the antibodies immobilized on a CM5 chip by the amine coupling method (Figure S1A). MEM-G9 and G233 were bound to the HLA-G1 monomer, while the 4H84 and MEM-G1 antibodies did not show any detectable binding at a concentration up to 50 nM (Figure S1A).

**Figure 1:**
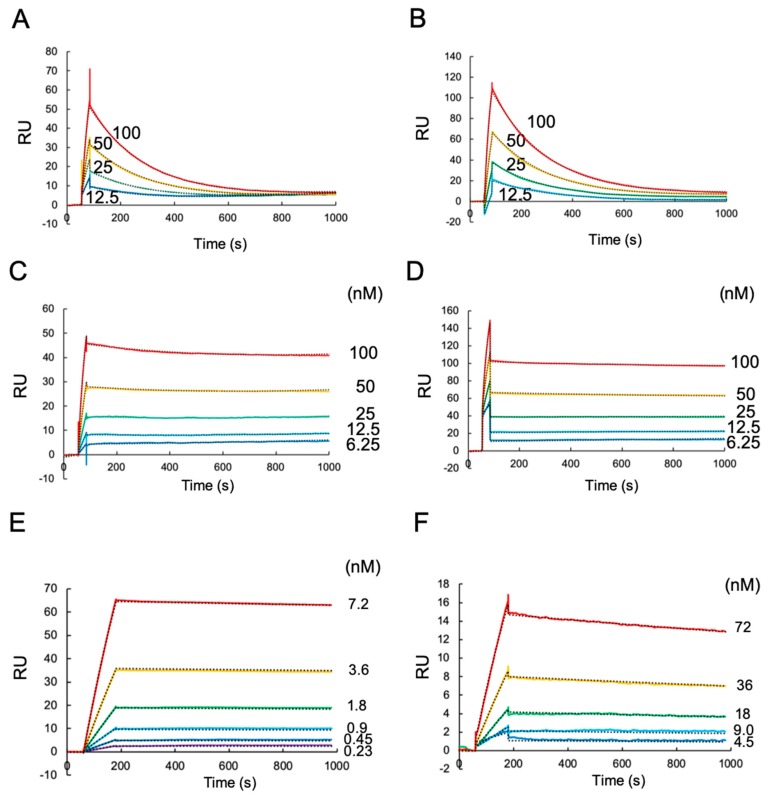
The surface plasmon resonance (SPR) of human leukocyte antigen (HLA)-G1 monomer, dimer, and HLA-G2 against each immobilized antibody: (**A**) 12.5–100 nM of the HLA-G1 monomer was injected against the immobilized MEM-G9, and (**B**) G233, respectively; (**C**) 6.25–100 nM HLA-G1 dimer was injected against the immobilized MEM-G9, and (**D**) G233, respectively; (**E**) 0.23–7.2 nM of HLA-G2 was injected against the immobilized 4H84; (**F**) 4.5–72 nM HLA-G2 was injected against the immobilized MEM-G1. The subtraction data of IgG control as the negative control are shown. Dash lines are the fitting curve of 1:1 binding model.

A one to one fitting model was applied to kinetics curve of the interaction of MEM-G9 and G233 with the HLA-G1 monomer, successfully determining the *K*_d_s of 15.1 ± 0.91 nM (global fitting, χ^2^ value is 0.05) and 13.2 ± 0.56 nM (global fitting, χ^2^ value is 0.31), respectively (Figure 1A,B).

Our previous study revealed that the dimer of HLA-G1, rather than the monomer, plays an important role in immune suppression via the avidity effect [23]. In order to investigate the reactivity of antibodies against the HLA-G1 dimer, the HLA-G1 dimer was injected into the immobilized antibody (Figure S1B). Similar to the reactivities against HLA-G1 monomer described above, MEM-G9 and G233 bound to the HLA-G1 dimer. The kinetic analysis revealed that *ka_1_*, *kd_1_*, *ka_2_*, and *kd_2_* of the interactions of the HLA-G1 dimer with MEM-G9 were determined as 1.54 × 10^5^ (1/Ms), 4.21 × 10^−4^ (1/s), 1.59 × 10^−4^ (1/RUs), and 4.36 × 10^−6^ (1/s), respectively. The *ka_1_*, *kd_1_*, *ka_2_*, and *kd_2_* of the interactions of the HLA-G1 dimer with G233 were also determined as 1.67 × 10^5^ (1/M·s), 8.24 × 10^−5^ (1/s), 7.94 × 10^−4^ (1/RU·s), and 2.58 × 10^−2^ (1/s), respectively. Apparent dissociation constants of the interaction of HLA-G1 dimer with MEM-G9 and G233 by using 1:1 binding model were 2.45 ± 0.32 nM (global fitting, χ^2^ value is 0.02) and 0.77 ± 0.11 nM (global fitting, χ^2^ value is 0.29), respectively (Figure 1C,D). 

### 2.2. Western Blotting and SPR Interaction Analyses of HLA-G2 Using the Antibodies

**Figure 2:**
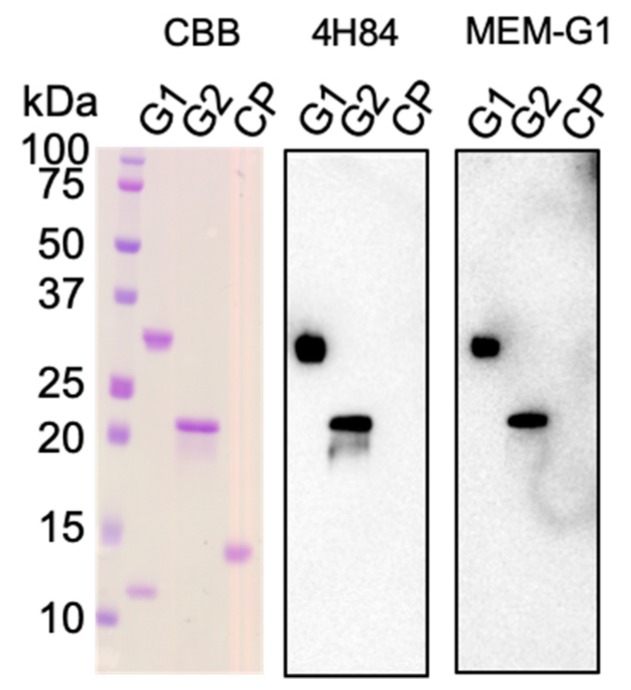
Coomassie brilliant blue (CBB) staining and Western blot analysis reacted with 4H84 and MEM-G1 of the HLA-G1 monomer and HLA-G2. The HLA-G1 monomer (G1), HLA-G2 (G2), and β2m, as a negative control protein (CP), were separated by Sodium dodecyl sulfate polyacrylamide gel electrophoresis (SDS-PAGE) in a reducing condition.

Previous studies demonstrated that MEM-G9 and G233 recognize native HLA-G proteins. In contrast, 4H84 and MEM-G1 recognize the denatured forms [26,27,33,34]. Here, we performed a Western blotting analysis on HLA-G1 and -G2 molecules. Figure 2 shows that both 4H84 and MEM-G1 have specific bands against HLA-G1, as well as HLA-G2, which does not contain the α2 domain, while MEM-G9 and G233 do not have such bands (data not shown). This result reveals that 4H84 and MEM-G1 recognize the sequential epitopes of either the α1 or α3 domains. Indeed, the 4H84 antibody was prepared by immunization using the synthetic peptide, DSDSACPRMEPRAPWVEQEGPEY, corresponding to a part (residues 61 to 83) of the HLA-G α1 domain. On the other hand, MEM-G1 was established via the immunization of the HLA-G1 extracellular domain, and its epitopes have not yet been determined. Consistently, SPR analysis demonstrated that, while HLA-G2 did not bind to MEM-G9 or G233, HLA-G2 showed specific and strong binding to 4H84 and MEM-G1 (Figure 1E,F and Figure S1C). These SPR and Western Blotting analyses suggest that the HLA-G2 molecule has an exposed and flexible part, which can be detectable for 4H84 and MEM-G1. 

### 2.3. The Competition Assay for the LILR Receptor Binding of HLA-G Isoforms with Anti-HLA-G Antibodies 

**Figure 3:**
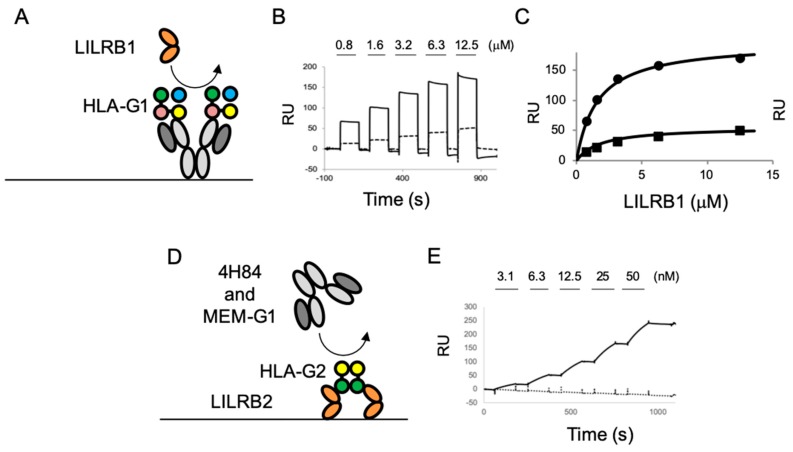
The competition assays of the MEM-G9 and G233 antibodies with leukocyte immunoglobulin-like receptor (LILR) B1, or 4H84 and MEM-G1 antibodies with LILRB2, using SPR. (**A**) Schematic image of the competition assay of the MEM-G9 and G233 antibodies with LILRB1. Yellow, pink, green and blue filled circles of HLA-G1 represent the domain α1, α2, α3, and β2m, respectively. (**B**) The sensorgram of LILRB1 injection (0.8–12.5 µM) against HLA-G1 immobilized by MEM-G9 (solid line) and G233 (dot line), with subtraction of the negative control (unimmobilized lane), are shown. (**C**) Equilibrium binding analysis of MEM-G9 (closed circle) and G233 (closed square). Solid lines represent nonlinear one to one fit for the Langmuir binding isotherm of each plot. (**D**) Schematic image of the competition assay of 4H84 and MEM-G1 antibodies with LILRB2. Yellow and green filled circles of HLA-G2 represent the domain α1 and α3, respectively. (**E**) The sensorgram of the continuous injection of 3.1–50 nM of 4H84 (solid line) and MEM-G1 (dot line) into the HLA-G2 immobilized by LILRB2, with subtraction of the negative control (HLA-G2 unimmobilized lane) are shown.

**Figure 4:**
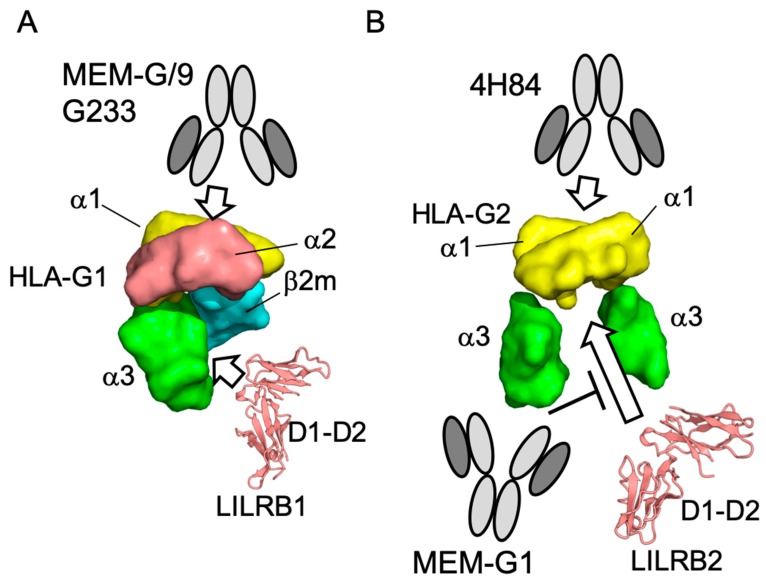
Schematic image of the epitopes of putative HLA-G antibodies and receptor ligand binding site of HLA-G isoforms. (**A**) The HLA-G1 binding site of MEM-G9 and G233 are not overlapped with those of LILRB1. (**B**) The binding site of MEM-G1, but not 4H84 on the HLA-G2 homodimer, is overlapped with that of LILRB2.

In order to further evaluate the antibody binding of HLA-G isoforms, we performed competition assays using the cognate receptors, LILRBs. The schematic images of the competition assays are shown in Figure 3A,D. The HLA-G1 dimer was injected into MEM-G9 or the G233 immobilized chip (Figure S2A). Then, LILRB1 was injected over the HLA-G1 dimer immobilized on the antibodies, showing that the LILRB1 bound HLA-G1 dimers were immobilized in both antibodies with concentration dependency (Figure 3B). The *K*_d_ values of the interaction between the LILRB1 and HLA-G1 dimers immobilized by MEM-G9 (1.5 μM) and G233 (2.5 μM) were similar to those of the interaction between LILRB1 and the immobilized HLA-G1 dimer (2.1 μM), as previously described (Figure 3C) [23]. These results indicate that the recognition site on HLA-G1 of LILRB1 is distinct from the epitopes of MEM-G9 and G233 (Figure 4A).

For the HLA-G2 competition assay, first, HLA-G2 was injected over the immobilized LILRB2 (Figure S2B). HLA-G2 was successfully immobilized with significant slow dissociation, indicating a remarkable avidity effect. MEM-G1 and 4H84 were injected over the HLA-G2 immobilized on LILRB2. MEM-G1 did not bind to LILRB2-captured HLA-G2, but 4H84 was bound (Figure 3E). These results revealed that the two antibodies have distinctly different epitopes compared to HLA-G2. Furthermore, this result suggests the possibility that the LILRB2-immoblized chip or well combined with 4H84 as a detection antibody could be utilized in a sandwich ELISA for HLA-G2.

## 3. Discussion

In this study, we showed that MEM-G9 and G233 bind to native HLA-G1 molecules but not to denatured HLA-G1 molecules or both the native and denatured forms of HLA-G2. In contrast, 4H84 and MEM-G1 bound to native HLA-G2, as well as the denatured forms of HLA-G1 and HLA-G2. 

One of the well characterized antibodies, MEM-G9, was developed against the human recombinant full length HLA-G1 protein [35]. Previous analyses indicated that MEM-G9 recognizes HLA-G1 expressing cells but has no binding activity to HLA-G2 expressing cells [26,27], which is consistent with our current study. In contrast, Pela et al. recently reported that immobilized native HLA-G2 was detected by MEM-G9 but not by MEM-G1 in ELISA, while the denatured HLA-G2 was bound to MEM-G1 but not to MEM-G9 [36]. They prepared HLA-G2 by the on-column refolding method, which is different from our procedure, and did not check any receptor-binding activities or structural characterization. On the other hand, the recombinant HLA-G2 prepared here has a functionally active form, which has the ability to bind to the receptors LILRB2 and the paired Ig-like receptor B (PIR-B), which is the mouse homolog of LILRB2 [9,17].

Moreover, our previous reports showed that HLA-G2 has an immune suppressive effect on collagen induced arthritis and atopic dermatitis-like skin model mice. [9,10]. Furthermore, Meiner et al. showed that HLA-G2 expressing cells bind to 4H84 but not to MEM-G9 [26], which is consistent with our present observations. 4H84 reportedly bound to the denatured form of the α1 domain of HLA-G1 by a mild acid treatment [37]. This result is consistent with the present Western blotting analysis, demonstrating that 4H84 bound to the denatured HLA-G isoforms. Notably, SPR analysis showed that 4H84 also bound to native HLA-G2. Indeed, 4H84 was produced by immunization of the peptide, which corresponds to 61–83 amino acid residues in the α1 domain of HLA-G isoforms [33]. The crystal structures of HLA-G1 revealed that these amino acid residues consist of an α helix, which contributes to the peptide binding together with α helix from the α2 domain [33]. In contrast, HLA-G2 does not include the α2 domain, and it is uncertain whether HLA-G2 has a binding ability towards peptides or any small molecules. Our previous structural analysis via electron microscope indicated that HLA-G2 has a relatively less homogenous structure with multiple conformations of the α3 domains, which might be caused by the flexible structure of the α1 domain [17]. Taken together, we propose that the α1 domain of HLA-G2 contains the flexible portion. On the other hand, the competition assay showed that the LILRB2 binding site of HLA-G2 overlaps with MEM-G1 but not 4H84, whose epitopes were not overlapped [28,31]. This result seems consistent with the idea we previously proposed, that the part of the α3 domain of HLA-G2 that faces β2m, when bound in the case of HLA-G1, is possibly involved in LILRB2 binding (Figure 4B) [17].

HLA-G is reportedly expressed in many types of cancer cells and involved in metastases [38]. Expression of the tolerogenic HLA-G molecule is considered to confer cancer cells to induce survival environment by suppressing the activation of immune cells. The importance of HLA-G1 molecules in cancer cells has become gradually understood. In contrast, other isoforms, such as HLA-G2, are still unclear [11]. This is due to the limited information on the specificity of HLA-G antibodies against each HLA-G isotype [11]. In this study, we found that the antibodies, 4H84 and MEM-G1, can detect native HLA-G2 but not native HLA-G1. Recently, immune checkpoint inhibitors, which block the interaction between the immune checkpoint receptor and its ligands, have become promising cancer therapeutic agents. Our competition assay revealed that MEM-G1 can block the interaction between HLA-G2 and LILRB2. Therefore, MEM-G1 could be a potential immune checkpoint inhibitor against the interaction between HLA-G2 and LILRB2.

In order to measure HLA-G molecules in autoimmune and cancer patients, HLA-G specific ELISAs were developed using monoclonal antibodies against HLA-G [24]. The antibodies MEM-G9 and G233 are widely used in HLA-G ELISA as a capture antibody [25]. Our SPR analysis revealed that ELISAs using MEM-G9/G233 are suitable for the detection of HLA-G1 molecules. In addition, it was also reported that an ELISA using a combination of antibodies 4H84 and MEM-G1 were suitable for the detection of soluble HLA-G [28]. Our present SPR analysis revealed that HLA-G2, not HLA-G1, molecules can be measured by ELISA using 4H84 and MEM-G1. This result suggests that these antibodies are useful for further functional analysis, as well as the determination of the precise amount of HLA-G2 molecules in vivo. Furthermore, our SPR competition assay revealed that 4H84 bound LILRB2-captured HLA-G2. Therefore, LILRB2, which is detectable for HLA-G2 and related isoforms, is a candidate protein for the capture molecule (instead of antibodies) in the development of ELISA.

In conclusion, we investigated the binding properties of the HLA-G antibodies MEM-G9, MEM-G1, G233, and 4H84 to an HLA-G1 dimer/monomer and HLA-G2. Furthermore, we performed competition experiments for the binding of the LILR receptor. These results provide important insight into the structural features of the HLA-G isotype and the quantitation of HLA-G isoforms for clinical diagnosis and therapy.

## 4. Materials and Methods

### 4.1. Antibodies

Commercially available anti-HLA-G monoclonal antibodies, MEM-G1 (IgG1), MEM-G9 (IgG1), G233 (IgG2a), and 4H84 (IgG1), from Abcam, were used in this study to characterize their binding activities to the HLA-G1 monomer and dimer and HLA-G2 (https://www.abcam.co.jp).

### 4.2. Production of the Ectodomains of HLA-G1 (Monomer/Dimer), HLA-G2, and LILRB Receptors

We previously reported the structures (either x-ray crystallography or electron microscopy) and receptor binding activities of the recombinant proteins used in this study were reported previously [9,17,23,39,40,41]. The HLA-G1 monomer and dimer, and HLA-G2, were produced by the same methods previously described [8,23,40]. Briefly, for HLA-G1 production, the α1-α2-α3 domains of the HLA-G heavy chain were expressed as the inclusion body in *Escherichia*. *coli*. After washing the inclusion body, the HLA-G’s heavy chains were refolded together with β2m and a peptide (RIIPRHLQL) via the dilution method. The refolded HLA-G1 was purified by gel filtration (Superdex75 26/60, GE) followed by anion exchange chromatography (Resource Q, GE) in the monomer form. The formation of the disulfide-bonded HLA-G1 dimer was proceeded by incubation of the purified HLA-G1 monomer (10 mg/mL), with 5 mM dithiothreitol (DTT) for 4 days at 4 °C. Finally, the HLA-G dimer was purified by gel filtration chromatography (Superdex 200 10/300, GE). For HLA-G2 production, the HLA-G2 ectodomain (the α1 and α3 domains of the heavy chain) was expressed as an inclusion body of *E. coli*, refolded by the dilution method and purified by gel filtration, similar to HLA-G1 proteins. LILRB1 and biotinated LILRB2 were also prepared by the same method previously described [23,42]. Briefly, N-terminal domains 1 and 2 of the extracellular domain of the LILR proteins were expressed as the inclusion body of *E. coli*, refolded by the dilution method and purified by gel filtration and anion exchange columns (Resource Q, GE).

### 4.3. SPR Analysis

Surface plasmon resonance (SPR) experiments were performed with Biacore T200 (GE). Then, 3.1–50 nM of the HLA-G1 monomer, dimer, or HLA-G2 proteins in HBS-EP were continuously injected over the immobilized antibodies and β2m (negative control), with a 10 µL/min flow rate. For the determination of the dissociation constant, 12.5–100 nM of HLA-G1 monomer and 6.25–100 nM of HLA-G1 dimer were injected against immobilized MEM-G9 and G233 and, 0.23–7.2 and 4.5–72 nM of HLA-G2 were injected against immobilized 4H84 and MEM-G1, respectively, with a 30 µL/min flow rate. The subtraction data of Ig control as the negative control were used for the calculation of kinetic parameters by bivalent and 1:1 fitting model. Dissociation constants were shown as average ± standard deviation of triplicate analyses.

The competition experiment was performed with the following procedures. For the HLA-G1 protein competition assay, MEM-G9 and G233 were immobilized on a CM5 chip. HLA-G1 was injected into the antibody immobilized lanes, followed by the injection of 0.8–12.5 µM of LILRB1. The dissociation constant between LILRB1 and the antibody immobilized HLA-G1 was determined by the equilibrium binding analysis previously described [23]. For HLA-G2, the biotinated LILRB2 was immobilized. Next, HLA-G2 was injected and captured. In total, 3.1–50 nM of 4H84 and MEM-G1 were injected over HLA-G2 on immobilized LILRB2.

### 4.4. Western Blotting

HLA-G1 and HLA-G2 were fractionated by sodium dodecyl sulfate polyacrylamide gel electrophoresis (SDS-PAGE) under a reducing condition and blotted against a nitro cellulose membrane. The 4H84 and MEM-G1 antibodies were used as primary antibodies, and an anti-mouse Fc with horse radish peroxidase (HRP) was used for the secondary antibodies.

## Supplementary Materials

The following are available at https://www.mdpi.com/1422-0067/20/23/5947/s1, Figure S1: Single cycle analysis of SPR of HLA-G1 monomer, dimer and HLA-G2 against immobilized each antibody. (A) HLA-G1 monomer and (B) HLA-G1 dimer from 3.1 nM to 50 nM continuously injected against immobilized G233 (bold line), MEM-G9 (dot line), MEM-G1 (solid line) and 4H84 (solid line). (C) HLA-G2 from 3.1 nM to 50 nM continuously injected against immobilized 4H84 (bold line), MEM-G1 (dot line), G233 (solid line) and MEM-G9 (solid line), Figure S2: Related to Figure 3 (A) and (C). (A) 100 nM of HLA-G1 dimer was injected against either MEM-G9 (black solid line), G233 (grey solid line) or β2m (black dot line, as a native control) immobilized on flow cell of BIAcore CM5 chip with 5 μL/min for 6 min. (B) 100 nM of HLA-G2 was injected against LILRB2 immobilized on flow cell with 5 μL/min for 10 min. The sensor gram of the injection of HLA-G2 against LILRB2 in advance of the injection of the antibody is shown in Figure 3C.

## Abbreviations

β2m	β2-microglobulin
CTLA-4	cytotoxic cell lymphocyte antigen-4
DTT	Dithiothreitol
ELISA	enzyme linked immunosolvent assay
HLA	Human Leucocyte antigen
HRP	horseradish peroxidase
LILR	Leukocyte immunoglobulin-like receptors
MHC	Main Histocompatibility Complex
PD-1	programed cell death protein-1
SDS-PAGE	sodium dodecyl sulfate polyacrylamide gel electrophoresis
SPR	Surface Plasmon Resonance
