# Supplementary material for: Evaluation of the Reactivity and Receptor Competition of HLA-G Isoforms toward Available Antibodies: Implications of Structural Characteristics of HLA-G Isoforms
Source: Int J Mol Sci. 2019 Nov 26;20(23):5947. doi: 10.3390/ijms20235947 (PMC6928721; doi:10.3390/ijms20235947)
Supplement: Supplementary file 1 [file ijms-20-05947-s001.pdf]

Supplementary Figures

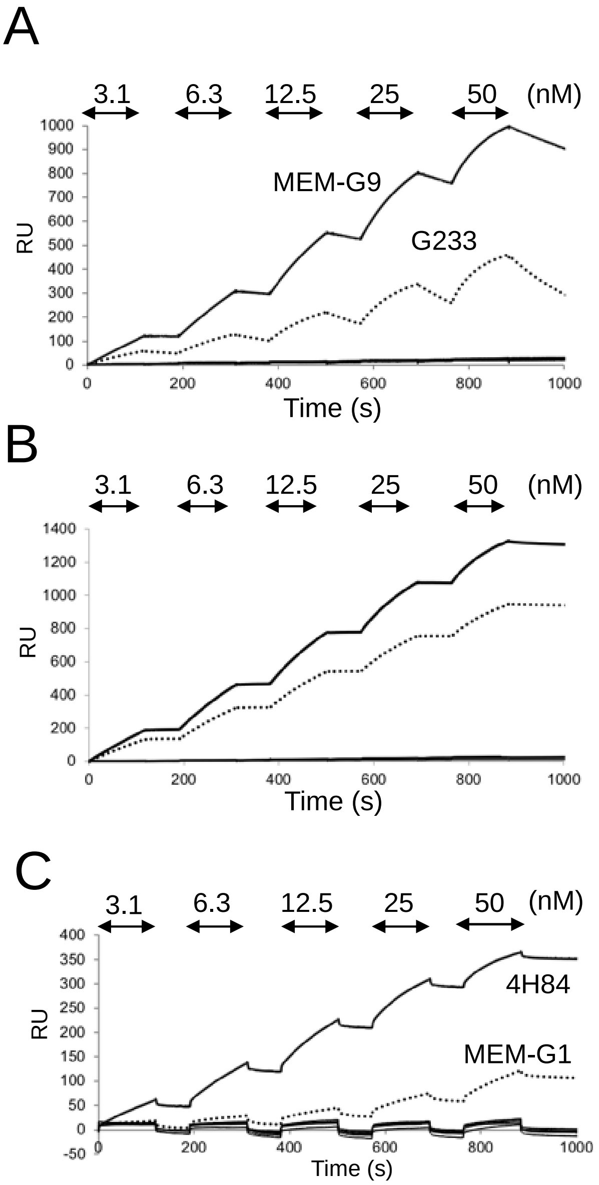

Figure S1. Single cycle analysis of SPR of HLA-G1 monomer, dimer and HLA-G2 against immobilized each antibody. (A) HLA-G1 monomer and (B) HLA-G1 dimer from 3.1 nM to 50 nM continuously injected against immobilized G233 (bold line), MEM-G9 (dot line), MEM-G1 (solid line) and 4H84 (solid line). (C) HLA-G2 from 3.1 nM to 50 nM continuously injected against immobilized 4H84 (bold line), MEM-G1 (dot line), G233 (solid line) and MEM-G9 (solid line).

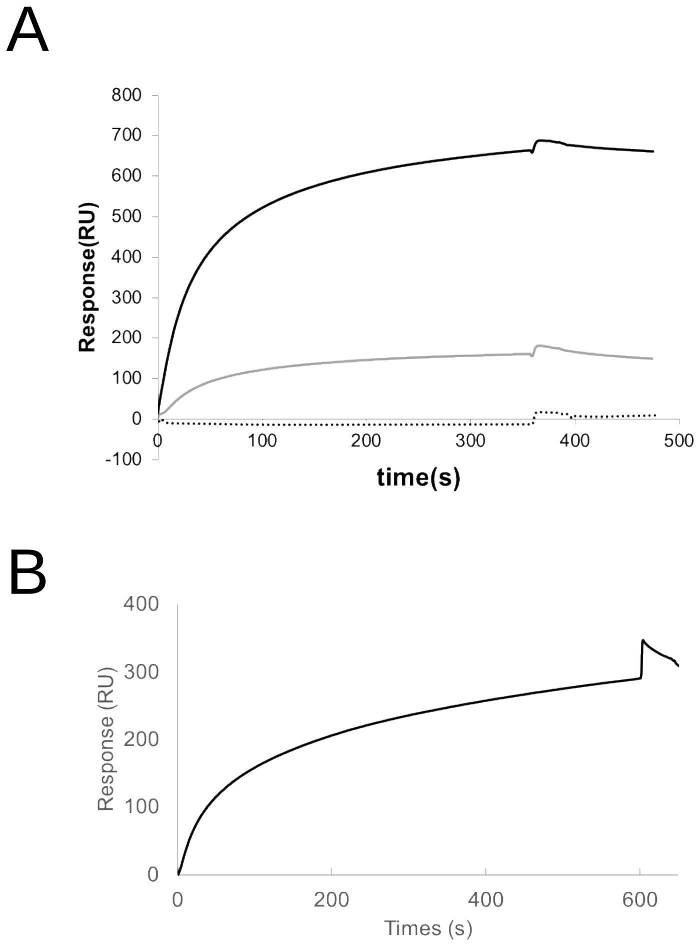

Figure S2. Related to Figure 3 (A) and (C). (A) 100 nM of HLA-G1 dimer was injected against either MEM-G9 (black solid line), G233 (grey solid line) or β2m (black dot line, as a native control) immobilized on flow cell of BIAcore CM5 chip with 5 μL/min for 6 min. (B) 100 nM of HLA-G2 was injected against LILRB2 immobilized on flow cell with 5 μL/min for 10 min. The sensor gram of the injection of HLA-G2 against LILRB2 in advance of the injection of the antibody is shown in Figure 3C.
